# Hemodynamic Forces Regulate Developmental Patterning of Atrial Conduction

**DOI:** 10.1371/journal.pone.0115207

**Published:** 2014-12-12

**Authors:** Michael C. Bressan, Jonathan D. Louie, Takashi Mikawa

**Affiliations:** Cardiovascular Research Institute, University of California San Francisco, San Francisco, CA, United States of America; Gent University, Belgium

## Abstract

Anomalous action potential conduction through the atrial chambers of the heart can lead to severe cardiac arrhythmia. To date, however, little is known regarding the mechanisms that pattern proper atrial conduction during development. Here we demonstrate that atrial muscle functionally diversifies into at least two heterogeneous subtypes, thin-walled myocardium and rapidly conducting muscle bundles, during a developmental window just following cardiac looping. During this process, atrial muscle bundles become enriched for the fast conduction markers *Cx40* and *Nav1.5*, similar to the precursors of the fast conduction Purkinje fiber network located within the trabeculae of the ventricles. In contrast to the ventricular trabeculae, however, atrial muscle bundles display an increased proliferation rate when compared to the surrounding myocardium. Interestingly, mechanical loading of the embryonic atrial muscle resulted in an induction of *Cx40*, *Nav1.5* and the cell cycle marker *Cyclin D1,* while decreasing atrial pressure via *in vivo* ligation of the vitelline blood vessels results in decreased atrial conduction velocity. Taken together, these data establish a novel model for atrial conduction patterning, whereby hemodynamic stretch coordinately induces proliferation and fast conduction marker expression, which in turn promotes the formation of large diameter muscle bundles to serve as preferential routes of conduction.

## Introduction

Whether the atrial musculature of the heart contains specialized tracts of cells capable of rapid electrical impulse propagation has been a topic of investigation for more than 100 years [1]–[7]. To date, a definitive fast conduction system, meeting the criteria first proposed by Aschoff and Mönckeberg [7]–[9] has yet to be identified in the atria [10]–[12]. Nonetheless, it is well documented that conduction though the atria is direction dependent, or anisotropic, with action potentials preferentially proceeding along the axial length of certain atrial muscle bundles [13]–[16]. Specifically, the terminal crest, a large muscle bundle adjacent to the venous entry of the heart, the pectinate muscles, a group of muscle bundles that branch from the terminal crest and insert into the vestibule of the tricuspid valve, and the bundle of Bachman, a collection of fibers that extend from the right to left atria, have been identified as preferential routes of conduction through the atria [13]–[21]. The apparent discrepancies between the lack of a histologically distinct conduction system in the atria and the clear delineation of preferential routes of conduction has led to some controversy between anatomists and electrophysiologists [6], [7], with the current consensus being that the orientation of the muscle fibers found within the atria provides a substrate for faster conduction routes. In turn, it has been suggested that the anisotropic nature of the atria can leave atrial myocardium particularly vulnerable to reentrant events leading to arrhythmia, especially in regions where bundles oriented in different directions overlap or branch [12], [18], [22]–[24].

Despite the importance of atrial conduction in both normal and pathological cardiac function, very little is known regarding the developmental mechanisms that pattern electrical impulse propagation through this region of the heart. In contrast, the formation of the ventricular conduction system (VCS) has been extensively investigated. Among the fundamental principles established for VCS specification, it has been noted that cells of the His bundle, bundle branches and peripheral Purkinje fiber network are clonally related to the working ventricular myocardium [25]–[27]. It has also been demonstrated that the recruitment of working myocardium into the VCS depends on hemodynamic-dependent and hemodynamic-independent endocardial endothelia-derived signals [28]–[30], and that myocardial cell recruitment into the VCS involves a decrease in proliferation rate [31], [32]. The observation that specific muscle bundles in the atria serve as preferential routes of conduction does suggest that some degree of specialization must take place among atrial myocytes during development, however, whether the atria and ventricles share similar mechanisms for fast conduction cell patterning remains unknown.

Several parameters determine the velocity at which an action potential can propagate through cardiac tissue. Broadly, these include the speed at which individual cells can depolarize and the resistance of the muscle fiber as a whole. The speed of depolarization depends on the kinetics of transmembrane current flow that is evoked upon stimulation, which is related to the types, density, and localization of voltage gated ion channels found within the sacrolemmic membrane of the cell [23], [33], [34]. Resistance, on the other hand, is proportional to the diameter of the muscle fiber, with increasing size providing a larger conduit for transmission, as well as cytoplasmic resistance and the conductance of cell-to-cell coupling via gap junctions. Consequently a fast conducting muscle fiber should contain cells capable of rapid depolarization, be of sufficient diameter to minimize overall resistance, and express high conductance gap junctions. Consistent with this, recent investigations into the development of atrial conduction has indicated that dramatic increases in action potential propagation speed occur concurrent with the formation of the large diameter pectinate muscles and Bachman's bundle [15], [35]. To date, it has yet to be determined how these muscle bundles are programmed to become sites of faster conduction.

This study, therefore, examined the physiological, morphological, and molecular changes that occur during muscle bundle formation in order to identify mechanisms that influence atrial conduction patterning. We demonstrate that molecular markers of fast conduction fate are sensitive to mechanical loading of the atria, suggesting that blood movement through the heart natively plays a critical role in regulating both atrial and ventricular conduction patterning. Unlike the ventricles, however, our data demonstrate that the patterning of fast conducting muscle bundles in the atria is associated with increased myocardial proliferation as opposed to the slower proliferation rate previously reported during VCS formation [31], [32]. These findings suggest a previously unrecognized mechanism for atrial conduction patterning in which hemodynamic stretch activates both cell proliferation and the expression of molecular components of fast conduction resulting in the formation of large diameter, fast conducting muscle bundles.

## Results

**Figure 1:**
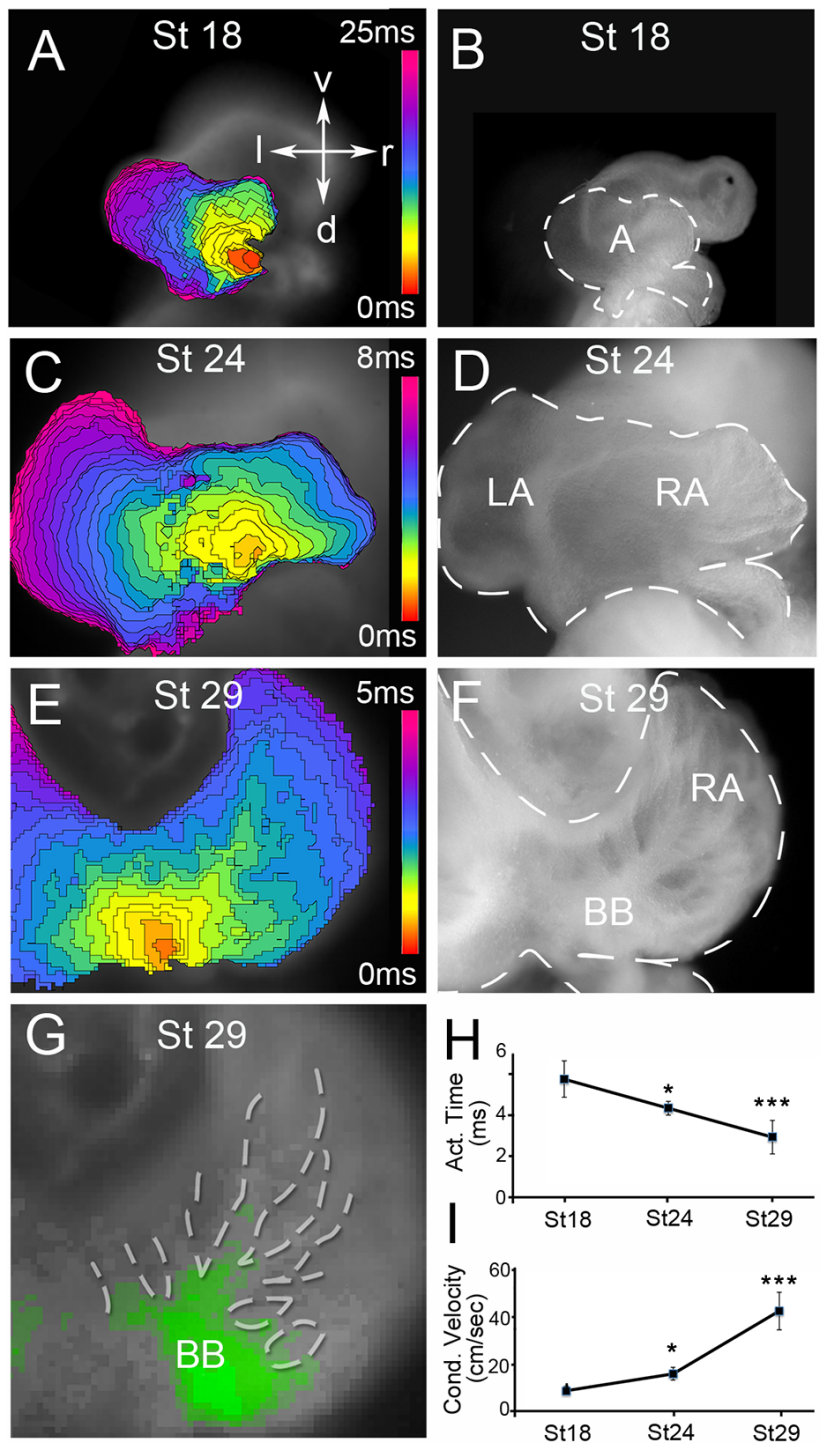
Developmental changes in atrial conduction patterning. **A**) Isochronal map (0.3 ms/div) depicting action potential propagation across the atria of a HH Stage 18 heart. The action potential initiates in the newly differentiated pacemaker cells (red) and propagates towards the left side of the atria (purple). **B**) Bright field image of the heart from (A), with the area of the atria and inflow region of the heart indicated by dashed lines. **C**) As in (A), for a HH Stage 24 atria. Note the emergence of finger-like projections in the isochronal map depicting regions of higher conduction velocity. **D**) Bright field image of heart from (C), with the area of the atria and inflow outlined by white dashed lines. **E**) As in (A), for a HH Stage 29 heart. Note the prominent finger-like extensions in the isochronal map demonstrating areas of rapid propagation. **F**) Bright field image of heart from (E), showing the location of the muscle bundles extending from Bachman's Bundle (BB) along the roof of the atria. **G**) Optical mapping data was superimposed on bright field images and the areas of the prominent muscle bundles were outlined (white dashed area) to demonstrate that rapid conduction followed the muscle bundles. Green signal  =  dV/dT max from a single frame of optical mapping recording. **H**) Quantification of average time for the right atria to activate at HH Stage 18, HH Stage 24, and HH Stage 29 (n = 8 for each). **I**) Quantification of right atrial conduction velocity from hearts in (H). P-values indicated significance relative to HH Stage 18 (***p<0.001, *p<0.05). v – ventral, l – left, d – dorsal, r – right, A - atria, LA – left atria, RA – right atria, BB – bundle of Bachman.

To identify the developmental window during which atrial conduction pathways form, we used optical mapping of voltage sensitive dyes [36]–[38] to construct high resolution maps (∼3,333 frames per second) of action potential propagation during the early stages of avian atrial morphogenesis. At HH Stage 18 [39], prior to muscle bundle formation, action potentials initiated in the newly differentiated cardiac pacemaker cells [36] entered the common atria at the sinoatrial junction (Fig. 1A), and proceeded along the right side of the common atria. In 9 of 11 hearts imaged, a region of action potential delay was present along the forming primary atrial septum at this stage, causing the right side of the common atria to activate prior to the left (Fig. 1A, S1 Movie). Importantly, no tracts of conduction were apparent in the atrial musculature at this stage with propagation through the right and left sides of the common atria occurring separately but uniformly. Following an additional 24 hrs of incubation to HH Stage 24, the atrial propagation pattern shifted dramatically. As at HH Stage 18, action potentials entered the atria at the sinoatrial junction, however, propagation across the right and left atria were no longer uniform (Fig. 1C). Instead action potentials followed tracts or preferential routes of conduction (Fig. 1C, S2 Movie). By HH Stage 29 these tracts were easily distinguishable, beginning adjacent to the sinoatrial junction and proceeding across the roof of the atria (Fig. 1E, S3 Movie). As it has previously been suggested that these tracts follow the pectinate muscle bundles [15], bright field images were overlaid with optical mapping data (Fig. 1G dashed area) to compare anatomical structures to routes of conduction. These data clearly demonstrate a correlation between the location of the large pectinate muscle bundles and preferential tracts of conduction in the atria. The formation of muscle bundles also correlated with increased overall atrial conduction velocity. At HH Stage 18, 5.75+/− 0.9 ms was required to activate the entire right atria. This was decreased to 4.35+/− 0.3 ms at HH Stage 24, and to 2.95+/− 0.8 ms by HH Stage 29 (Fig. 1H). This decrease in activation time occurred despite an increase in atrial area across these stages and was the result of a dramatic increase in conduction velocity from 7.1+/− 1.9 cm/sec at HH Stage 18 to 40.0+/− 4.6 cm/sec at HH Stage 29 (Fig. 1I). These data indicate that atrial conduction patterning transitions from uniform to fast conducting pathways along the pectinate muscle bundles between HH Stages 18–29.

**Figure 2:**
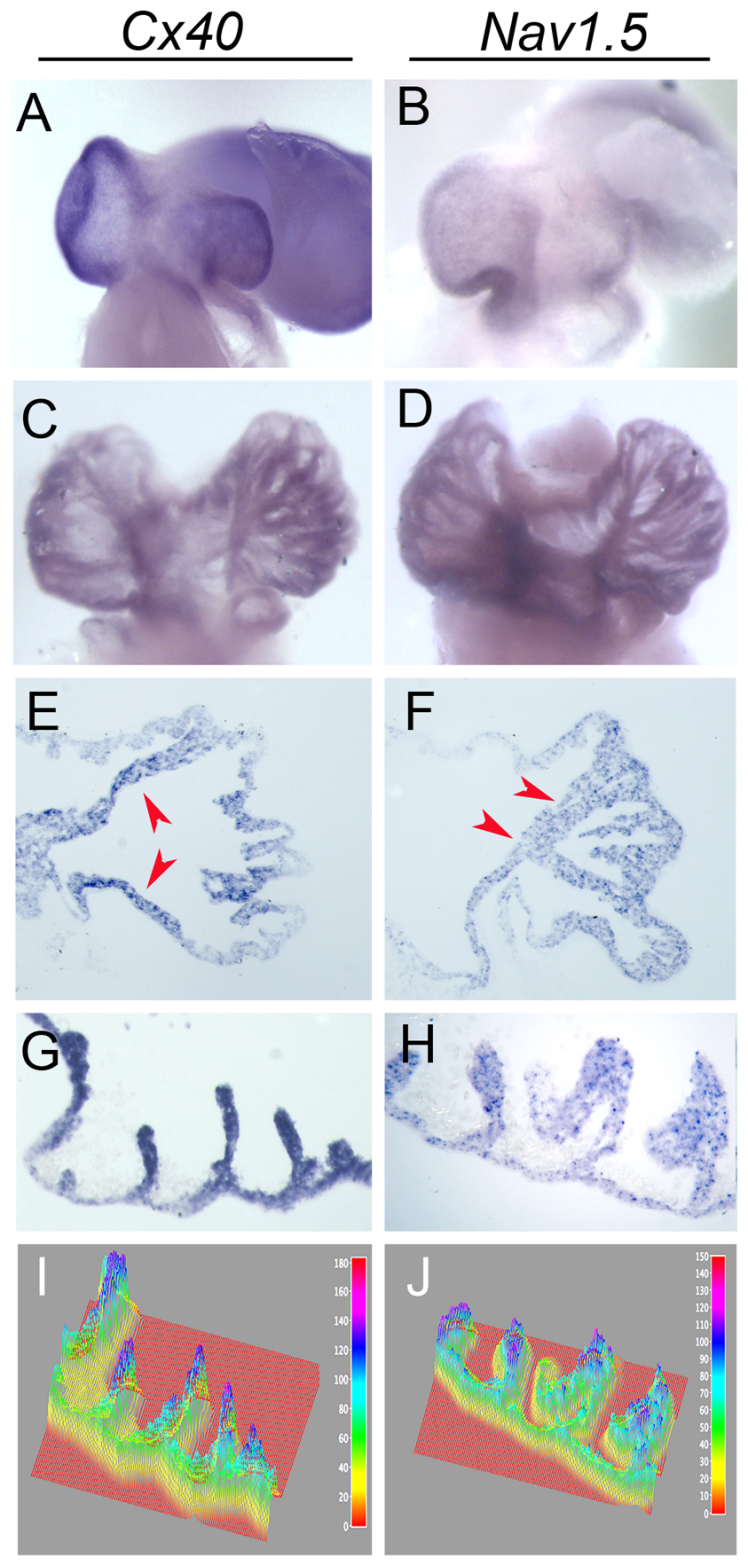
Molecular diversification of atrial muscle bundles. **A,B**) Whole mount *in situ* hybridization for *Cx40* and *Nav1.5* at HH Stage 18. Viewed from a superior angle of the atrial roof. **C,D**) Whole mount *in situ* hybridization for *Cx40* and *Nav1.5* at HH Stage 29. Note deep staining along the muscle bundles. **E,F**) *In situ* hybridization for *Cx40* and *Nav1.5* performed on transverse sections through the roof of the atria. Note that *Cx40* and *Nav1.5* are enriched in the prominent pectinate muscle bundles (red arrows). **G,H**) *In situ* hybridization for *Cx40* and *Nav1.5* performed on transverse sections inferior to the atrial roof. Note deeper staining in the muscle bindles than in the thin-walled myocardium. **I,J**) Plots of staining intensity from sections in (G,H). Note that the relative intensity of the thin-walled myocardium (yellow/green) is lower than the myocardium at the tips of the muscle bundles (blue/purple).

To determine if these fast conducting tracts of atrial myocytes displayed molecular characteristics distinct from the adjacent myocardium, we examined the expression of conduction markers by *in situ* hybridization. At HH Stage 18, *Cx40,* a well described marker of fast conducting cell populations in the heart [26], was distributed evenly across the right and left sides of the common atria, with a region of low *Cx40* expression adjacent to the forming primary atrial septum (Fig. 2A). This pattern was consistent with the propagation pattern described in the above optical mapping studies (compare Fig. 1A with Fig. 2A). By HH Stage 29, whole mount and section *in situ* hybridization demonstrated that *Cx40* was enriched in the atrial muscle bundles in a pattern consistent with tracts of action potential propagation noted in Fig. 1E (Fig. 2C,E,G,I). Similar to *Cx40*, mRNA for *SCN5a,* which encodes for the rapidly activating voltage gated sodium channel, Nav1.5, was expressed uniformly across the roof of the HH Stage 18 atria (Fig. 2B). However, by HH Stage 29 *Nav1.5* also displayed enrichment in the atrial muscle bundles when compared with the thin-walled myocardium (Fig. 2D,F,H,J). These data demonstrate that as atrial muscle bundles become functionally diverse from the thin-walled myocardium, they become enriched for *Cx40* and *Nav1.5*.

It is well documented that a similar enrichment of *Cx40* and *Nav1.5* occurs during VCS differentiation [26], [29], [40]. At HH Stage 29 both *Cx40* and *Nav1.5* are upregulated in the ventricular trabeculae when compared to the compact myocardium (S1 Figure). The similarities between early atrial and ventricular patterning led us to examine the expression of late markers of the VCS in atrial tissue. The localization of a slow twitch myosin heavy chain (sMHC) previously associated with Purkinje fiber differentiation in avians [26] was therefore examined. At HH Stage 29, sMHC was not detectable in either atrial muscle or ventricular muscle. However, at HH Stage 40 and beyond, sMHC was found in the subendocardial and perivascular myocytes of the atrial muscle bundles (S2 Figure C), which was consistent with its subendocardial and perivascular localization in the ventricular Purkinje fibers (S2 Figure E). Collectively, these data indicate that atrial myocardium diverges both functionally and molecularly as atrial morphogenesis progresses leading to the establishment of specialized preferential routes of conduction.

**Figure 3:**
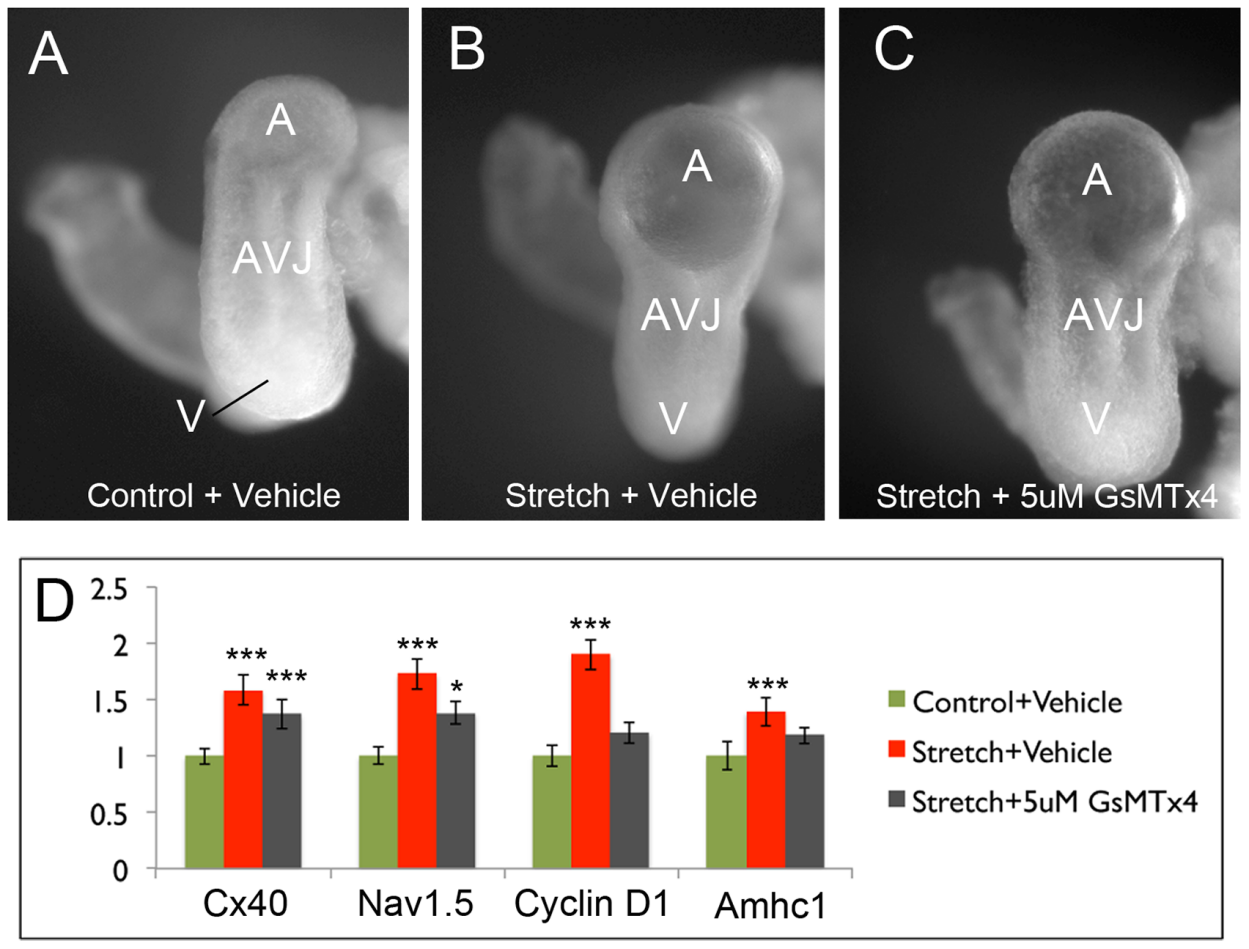
Stretching induces conduction marker expression in the atria. Whole embryonic hearts were isolated at HH Stage 18 and placed in culture for 8 hrs. **A**) Image of control heart (left lateral view) following 8 hrs of culture with vehicle (Tyrode's solution). **B**) Image of heart in which a bead of silicone oil was microinjected into the atria following 8 hrs. of culture with vehicle. **C**) Image of silicone injected atria cultured for 8 hrs with 5 uM GsMTx4. **D**) Quantification of gene expression levels in atria following whole heart culture. P-values indicated significance relative to control (***p<0.001, *p<0.05). A – atria, AVJ – atrioventricular junction, V – ventricle.

The above data led to the question of how the diversification of atrial myocytes between HH Stages 18–29 is regulated. The formation of the peripheral VCS has been shown to require recruitment of working myocardium into a fast conducting Purkinje fiber fate. At least one hemodynamic-dependent signaling axis has been identified to activate Purkinje fiber specification [28], [29]. Based on the molecular similarities between the atria and ventricles described above, we examined if analogous mechanisms might influence atrial conduction. To assess whether hemodynamics play a role in the regulation of atrial conducting fate, HH Stage 18 hearts were isolated and placed in culture for 8 hrs under control conditions or following mechanical loading of the atria through microinjection of a bead of silicone oil (Fig. 3A-C) [41]. The silicone oil prevented the atria from collapsing during diastole, in effect maintaining constant stretch throughout the cardiac cycle. Stretch treatment led to a minor, albeit significant (1.3 fold, p<0.01) induction of the structural gene Atrial Myosin Heavy Chain 1 (*AMHC1*), and a more pronounced induction of *Cx40* and *Nav1.5* (1.6 and 1.7 fold, respectively) when compared with the control condition (Fig. 3D). Importantly, treatment of the stretched atria with 5 uM GsMTx4, which blocks stretch activated ion channel activity by changing membrane tension [42], attenuated increases in *Cx40* and *Nav1.5* expression (Fig. 3D). These data demonstrate that mechanical loading of the naïve atria triggers a stretch-dependent induction of *Cx40* and *Nav1.5* expression.

As the fast conducting ventricular Purkinje fibers are thought to exit the cell cycle shortly after their lineage segregation, the proliferation marker, *Cyclin D1,* [43], [44] was also examined in stretched atria. Surprisingly, however, when compared to control atria, stretch induced a 2.1 fold increase in *Cyclin D1* expression, which was also attenuated by introduction of GsMTx4 (Fig. 3D). Interestingly, these findings suggested that, unlike the ventricles, increased proliferation rate may be a hallmark of fast conducting regions of the atria.

**Figure 4:**
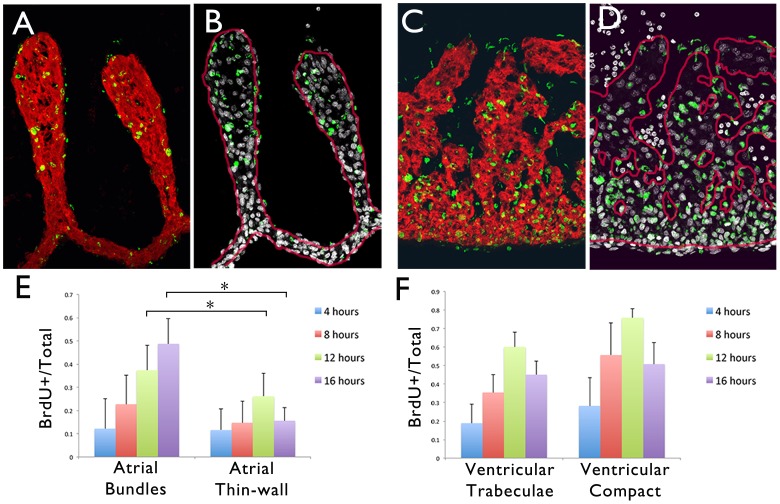
Atrial muscle bundles display increased proliferation rate relative to thin-walled myocardium. **A**) Immunohistochemical staining in HH Stage 29 atrial muscle pulsed for 12 hrs with BrdU using antibodies against sarcomeric myosin (MF20-red) and BrdU (green). **B**) Section from (A) showing overlap of BrdU (green) and the nuclear marker Dapi (grey), with the MF20 positive area from (A) outlined (red line). **C,D**) As in (A,B) for the ventricular trabeculae. E) Quantification of BrdU incorporation within atrial muscle bundles (thicker then 4 nuclei) and thin-walled myocardium (4 nuclei or less). *p<0.05. F) Quantification of BrdU incorporation within ventricular trabeculae (thicker than 4 nuclei) and compact myocardium (4 nuclei or less).

The above data would predict that areas of highest *Cx40/Nav1.5* expression in the atria (i.e. the pectinate muscle bundles) should also display the highest native proliferation rate. To test this, HH Stage 29 embryos were pulsed *in ovo* with BrdU for periods of 4 hrs, 8 hrs, 12 hrs, or 16 hrs to determine the relative proliferation rates of the atrial muscle bundles compared to the thin-walled myocardium (Fig. 4). At HH Stage 29, a gradient of proliferation, highest in the compact myocardium to lowest in the trabeculae was noted. This trend was consistent for all pulse lengths examined, but did not reach statistical significance at this stage (Fig. 4C,D,F). Conversely, at all pulse lengths examined, BrdU incorporation into the myocardium of the atrial muscle bundles trended higher than the thin-walled myocardium reaching statistical significance for the 12 and 16 hr pulses (Fig. 4A,B,E). These data confirm that the areas of highest *Cx40/Nav1.5* expression in the atrial muscle also displayed the highest proliferation rate.

**Figure 5:**
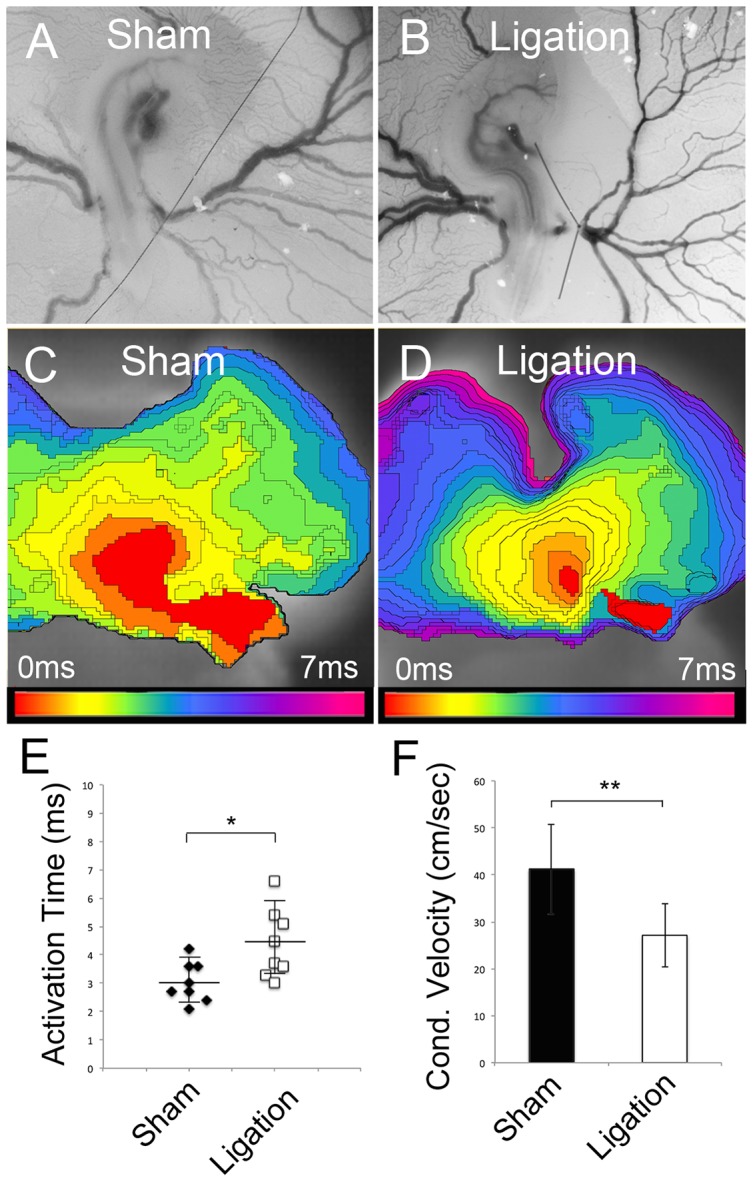
Right vitelline vessel ligation decreases atrial conduction velocity. **A**) Bright field image of sham operated embryo at HH Stage 18. Suture was placed under the vitelline artery and vein but was not tied. **B**) Bright field image of vitelline vessel ligation in a HH Stage 18 embryo. **C**) Isochronal map (0.3 ms/div) of HH Stage 29 sham operated embryo viewed from a superior angle to capture the roof of the right atria. Action potentials propagated from the sinoatrial junction (red) to ventral extent of the right atria (blue). **D**) Isochronal map of action potential propagation in an embryo at HH Stage 29 following vitelline vein ligation. Note that the distance between adjacent isochronal divisions is decreased, demonstrating slower conduction velocity. **E**) Quantification of right atrial activation time in sham operated (black diamonds) vs. ligated embryos (white boxes). n = 8, *p<0.05. **F**) Quantification of conduction velocity in sham operated (black bar) vs. ligated embryos (white bar). n = 8, **p<0.01.

The ability of biophysical stretch to induce *Cx40/Nav1.5* and *Cyclin D1* expression, coupled to the co-localization of conduction system markers with areas of increased proliferation in the fast conducting muscle bundles, led us to examine whether atrial pressure was a functional regulator of conduction velocity *in vivo*. To test this, the volume of blood returning to the heart was decreased via ligation of the right vitelline vessels around HH Stage 18, prior to muscle bundle formation (Fig. 5A,B). This procedure has previously been shown to decrease stroke volume and blood flow [45], and in our hands resulted in a dramatic regression of the vasculature on the right side of the embryo within 24 hrs (S3 Figure). Following ligation, we allowed sham operated and ligated embryos to develop to HH Stage 29, when preferential routes of fast conduction should be present, and then optically mapped the atria to examine conduction characteristics. It should be noted that by HH Stage 29, three days post ligation, the vasculature around the ligation site had remodeled allowing for relatively normal return of blood to the heart (S3 Figure). Significant differences in conduction characteristics were present between sham operated and ligated embryos at HH Stage 29 (Fig. 5C–F). In sham operated embryos the total time required to activate the right atria was 3.10+/− 0.6 ms (Fig. 5E). The time required to activate the right atria in ligated embryos had, by comparison, increased to 4.51+/− 1.2 ms (Fig. 5E). The increase in the time required to activate the right atria was due to a slowing of conduction velocity from 41.2+/− 9.5 cm/sec in sham atria to 27.1+/− 6.9 cm/sec in ligated embryos (Fig. 5F). These data demonstrate that decreasing atrial stretch by restricting blood return to the heart results in slowing atrial conduction velocity. Based on these results our data collectively suggest that atrial stretch is a critical regulator of the functional and molecular diversification of preferential routes of conduction during atrial development.

## Discussion

The specialized cells of the VCS have been characterized based on their distinct histological appearance [46], the presence of insulating connective tissue separating these cells from the working myocardium [11], and by the unique molecular expression profile that they exhibit [26], [29], [40], [47]–[49]. To date, a cell population in the atria with analogous features to the VCS has not been identified. Nonetheless, anisotropic propagation through mature atrial tissue has been reported in multiple species [13], [15], [20], [50]–[53], with preferential routes of conduction proposed to coincide with the developing pectinate muscle bundles along the atrial roof [15], [35]. It is believed that the structural characteristics of these muscle bundles allow for their faster conduction properties. Specifically, the large cross-sectional area of the muscle bundles creates low resistance conduits for conduction, and the orientation of gap junctions (preferentially in line with the axis of the muscle bundles) establishes anisotropic propagation. However, how atrial conduction is patterned during development has remained poorly understood. Here we have demonstrated that during a developmental window spanning HH Stage 18 to HH Stage 29 in avians, atrial muscle becomes both functionally and molecularly heterogeneous. Importantly, this heterogeneity manifests as the emergence of thick muscle bundles that serve as preferential routes of action potential conduction through the atrial tissue. Consistent with the formation of the VCS, we demonstrate that atrial muscle bundles become enriched for the expression of the low resistance gap junction, *Cx40,* and the rapidly activating voltage gated sodium channel, *Nav1.5,* during early cardiogenesis. Of note, our HH Stage 29 atrial *Cx40* expression data are consistent with the localization of GFP recently reported in the Cx40:eGFP knock in mouse [35], [54], however, to our knowledge, *Nav1.5* has not previously been described as being upregulated in the bundles. We examined these markers specifically due to their previously identified expression within the ventricular conduction system [26], [29], [40]. The enrichment of both *Nav1.5* and *Cx40* in the faster conducting muscle bundles indicates that these regions adopt a phenotypic fate distinct from the thin-walled atrial myocardium. While we have previously investigated the expression of two additional cardiac gap junctions, *Cx43* and *Cx45* [55], noting that both are expressed at much lower levels than *Cx40* at stages just prior to muscle bundle formation, future studies will be required to address whether these gap junctions also regionally segregate during atrial development. Additionally, it has long been known that the excitability of myocytes changes dramatically during cardiac development [56], [57], however, it remains to be determined whether ion channels in addition to Nav1.5, such as voltage gated calcium channels and/or potassium channels are distributed unevenly between muscle bundles and thin-walled atrial myocardium.

The functional and molecular differences observed between the atrial muscle bundles and the thin-walled myocardium led us to investigate potential epigenetic factors that might regulate the establishment of preferential routes of conduction. One model for VCS development stipulates that increases in hemodynamic forces, including pressure/stretch, induce endocardial-derived signaling factors, which in turn, activate the expression of fast conducting markers in myocytes that are in close proximity to the endocardium [26], [29]. Consistent with this model, upregulation of conduction system markers is particularly evident in the trabeculae, connected sheets of myocardium that extend into the ventricular lumen. The atrial muscle bundles display some anatomical similarities to ventricular trabeculae in that they are thicker ridges of muscle that extend into the lumen of the heart, sharing a large surface area with the endocardium. Based on the molecular and anatomical similarities, we tested whether changes in hemodynamics would influence atrial conduction by microinjecting silicone oil into the atria of HH Stage 18 hearts. Importantly, atrial propagation at this stage was uniform, suggesting that the events required for diversification of conduction had not yet occurred. Following 8 hrs of culture, hearts stretched with the silicone oil [41] displayed increased expression of *Cx40* and *Nav1.5* when compared with control hearts. This suggests that the atria display a similar sensitivity to pressure/stretch to what has previously been noted in the ventricles [29]. Interestingly, stretch also induced a slight increase in the expression of *AMHC1*, which encodes for a Beta-myosin heavy chain expressed in the embryonic atria [58], [59]. Based on the induction of *AMHC1* we cannot rule out that stretching the atria resulted in a general hyperplastic response, however, it should be noted that the magnitude of the *AMHC1* induction was smaller than the conduction genes examined.

It was unexpected that atrial stretch resulted in the co-induction of *Cyclin D1* with *Cx40* and *Nav1.5* in our *ex vivo* culture system. We therefore examined whether proliferation was most prevalent in the regions of the atria that natively displayed the highest expression of *Cx40* and *Nav1.5*, i.e. the muscle bundles. Surprisingly, BrdU incorporation demonstrated that the atrial muscle bundles had a higher proliferation rate than the adjacent thin-walled myocardium. This is a major departure from fast conduction patterning in the ventricle where the recruitment of working myocardium into the VCS network is thought to involve a decrease in cell proliferation rate [25], [28], [31], [32]. This suggests that while stretch is a positive regulator of a faster conduction phenotype in both atria and ventricle, the process that ultimately induces muscle bundle formation is not completely analogous to ventricular trabeculae development. Since other gap junctional proteins have previously been identified as positive or negative regulators of cell cycle progression [60]–[63], it is possible that *Cx40* and/or *Nav1.5* are causally related to the increased proliferation rate in the atrial bundles. However, it should be noted that the proliferation index between the *Cx40/Nav1.5* positive fast conducting regions of the atria and ventricles were inverted suggesting that this relationship may be associative in the atria. A definitive answer to the issue will require future studies.

When isolated and placed under our culture conditions, embryonic hearts do not complete morphogenesis. As such, it was not possible to determine the ability of stretch to influence conduction velocity in our *in vitro* silicone oil assay due to incomplete atrial development. Therefore, we selected an *in vivo* vascular ligation model, previously reported to decrease blood flow through the heart [45], to test the effects of hemodynamics on the long term patterning of atrial conduction. Ligation of the vitelline vessels, in our hands, resulted in a dramatic decrease in vascular area 24 hrs following surgery. It should be noted that the procedure likely had additional effects beyond simply limiting blood return to the heart. However, the vasculature around the ligation site remodeled within 48 hrs, allowing for relatively normal overall development. Consequently, conduction patterning could be examined at advanced stages of atrial morphogenesis. Importantly, ligation resulted in a 34% drop in conduction velocity when compared with sham operated embryos. For comparison, this drop in conduction velocity fell between the values obtained from homozygous Cx40−/− (67% drop in conduction velocity) and heterozygous Cx40+/− (31% drop in conduction velocity) knockout mice at E12.5 [35]. This demonstrates that a relatively subtle change in blood flow has a significant effect in conduction velocity, suggesting that hemodynamic stretch plays a critical role in atrial conduction patterning.

These findings led to a model by which increased hemodynamic stress during development leads to an adaptive response resulting in increased conduction velocity in the atria. Several major questions, however, remain to be addressed. Among these, it is still unclear how hemodynmic stresses are translated such that a regionalized pattern of muscle bundles emerges. Furthermore, it remains to be determined which cell-type, myocardium and/or endocardium, is principally responsible for sensing stretch in the atria.

In conclusion, the current study has identified that the developing atrial muscle adopts a system for fast conduction propagation unique from the VCS. Instead of specifying an insulated Purkinje fiber system, morphologically homogenous atrial muscle segregates into at least two distinct regions, muscle bundles enriched for the low resistance gap junction *Cx40* and the rapidly activating sodium channel *Nav1.5*, and a slower conducting thin-walled myocardium. In effect, this results in the formation of large diameter conduits of action potential propagation, capable of rapid depolarization and ion transmission to adjacent cells. Therefore, the current study reconciles previous controversies between anatomists and physiologists surrounding the presence of a specialized atrial conduction system, demonstrating that there is a regionalized diversification of cell types that occurs during atrial development leading to the formation of a faster conduction network.

## Materials and Methods

### Chicken Embryos

White Leghorn Horn Chicken eggs were obtained from Petaluma Farms (Petaluma, CA) and incubated at 38C° to desired stages.

### Optical mapping of voltage sensitive dyes

Procedures for optical mapping have been described previously [36], [55]. All imaging was performed at 37.7+/− 0.7°C in staining solution (Tyrode's solution, 10 mM Hepes, 12 mM NaHCO_3_, 10 uM Di-4-ANEPPS, and 20 uM Cytochalasin-D [Sigma], pH 7.4) saturated with 95% O_2_/5% CO_2_. Immediately following optical mapping hearts were fixed in 4% PFA overnight at 4°C. They were then transferred to a Leica MZ16 F stereo microscope and bright field images were acquired to compare conduction routes to anatomical structure. Isochronal maps were constructed from image sequences captured at 0.3 ms intervals using BV Analysis software V13.12.20 (BrainVision Inc, Tokyo, Japan). Conduction velocity was calculated as the change in distance over change in time for the dV/dT maximum along the vector of propagation through the right atria.

### In situ Hybridization

Procedures for *in situ* hybridization have been described previously ([1], [36], [64]. The relative intensity of *Cx40* and *Nav1.5* staining was determined using ImageJ (V1.48). Following *in situ* hybridization, transverse sections through the atria were photographed and images were imported into ImageJ (V1.48). These images were then grey-scaled and inverted. The relative intensity of each pixel in the image was then calculated and plotted.

### In vitro heart explantation

HH Stage 18 hearts were isolated and cultured as previously described [55]. Briefly, the inflow, atria, atrioventricular junction, ventricle and 3/4 of the outflow tract were isolated and transferred to DMEM/F12 high glucose media supplemented with 1% penicillin/streptomycin. Hearts were then cultured for 8 hrs at 37°C in 95% O_2_/5% CO_2_. To stretch atrial myocardium, silicone oil was microinjected into isolated hearts prior to culture. Injection needles backloaded with silicone oil (GFS Chemical, 8297) were fashioned from borosilicate glass capillaries (World Precision Instruments, Inc., KTW100-3) with a vertical pipette puller (David Kopf Instruments, Model 720). A small incision was made where the right common cardinal vein meets the sinus venosus, and the micropipette was passed through this incision into the lumen of the atria. A pressure injector (Eppendorf, Femtojet) was then used to deliver a bead of silicone oil into the atrial lumen (beads were ∼512 um in diameter). Following 8 hrs of culture atria were isolated and RNA extraction was performed. To block stretch activated responses hearts were cultured with 5 uM GsMTx4 (Alomone Labs, STG-100) dissolved in Tyrode's solution (pH 7.4).

### Real-Time PCR

RNA was isolated using RNAeasy kits (Qiagen), and cDNA was synthesized using iScript Select (Biorad) according to manufacturer's instructions. Real-time PCR was performed on a Step-One Plus thermocycler (Applied Biosystems). For relative expression CT values were standardized to *Gapdh* and fold changes were calculated using the deltadelta CT method. Primer sequences were as follows: Gapdh Forward: 5′-CCCCCAATGTCTCTGTTGTT-3′, Gapdh Reverse: 5′-CATCCAAGGTGGAGGAATGG-3′, Nav1.5 Forward: 5′-TCCATCCAGTCAGAAGAGCA-3′, Nav1.5 Reverse: 5′-GAGTCTCTGATTGT GCCATG-3′, Cyclin D1 Forward: 5′-CACTTGGATGCTGGAGGTCTG-3′, Cyclin D1 Reverse: 5′-GCACAGTTTTTCTGCGGTCA-3′, Cx40 Forward: 5′-CACA GCTGCCTTGTGTCTAA-3′, Cx40 Reverse: 5′-CAACCAGTCATCTGAGCGAT-3′, AMHC1 Forward: 5′-GCCTATAAGAGGCAGGCAGA-3′, AMHC1 Reverse: 5′- GAGACTCAGCCATGTCAGCA-3′.

### BrdU Labeling

Chick embryos were placed in shell-less culture at HH Stage 18. Briefly, embryos and yolk were transferred into square polystyrene weigh boats (VWR, 89106-766) and covered with cell culture dish lids (Cellstar, 639160). Embryos were then incubated at 38 C° to HH Stage 29. A small incision was then made through the amniotic membrane and 50 ul of BrdU (10 mM, Life technologies, B23151) was pipetted over the trunk of the embryo. Following 4 hrs, 8 hrs, 12 hrs, and 16 hrs, hearts were fixed for 30 min in 2% PFA and prepared for immunohistochemistry.

### Immunohistochemistry

For sMHC labeling: 12 um thick cryosections were air dried and washed in 1× PBS (pH 7.4), 3×5 min. Autofluorescence was quenched by a 30 min treatment in freshly made 0.1 M glycine (1× PBS, pH 7.4). Antigen retrieval was then performed by treating slides in freshly made 0.01 M sodium citrate buffer (dH2O, 0.05% Tween 20, pH 6, preheated to 95°C) for 13 min at 95°C. Slides were then blocked for 1 hr in Blocking solution 1 (1× PBS [pH 7.4], 1% BSA, 10% goat serum, 0.1% Triton X-100) at room temperature. During this time, the primary antibody for sMHC (ALD58, Developmental Studies Hybridoma Bank) was diluted (1∶50) in Blocking solution 2 (1× PBS [pH 7.4], 1% BSA, 10% goat serum, 0.1% Tween 20) and spun for 5 min at 15,000 rpm. 300 ul of supernatant was then carefully pipetted onto slides, which were incubated overnight at 4°C. Negative controls were incubated with Blocking solution 2 only. Slides were then washed in 3× PBS (pH 7.4), 3×5 min. Secondary antibody (Alexa 594) were diluted in Blocking solution 2 at 1∶200, and slides were incubated for 1 hr at room temperature in the dark. Slides were washed in the dark in 3× PBS (pH 7.4), 3×5 min. Coverslips were then mounted with Vectashield Mounting Medium for Fluorescence with DAPI (Vector Laboratories, Inc., H-1200) and stored at 4°C until imaging. For BrdU and MF20 co-labeling, the protocol was the same as above except for the following: between the initial 1× PBS and 0.1 M glycine washes, in lieu of antigen retrieval, slides were first moved to pre-warmed 37°C 1× PBS (pH 7.4) for 20 min at 37°C. Tissue was then placed in pre-warmed 37°C 1 M HCl and incubated for 15 min at 37°C to facilitate antibody access to DNA. This was followed by a 10 min wash in freshly made 0.1 M sodium tetraborate at room temperature, and proceeded by a 3×5 min wash in 1× PBS (pH 7.4). Slides were then incubated with an anti-BrdU antibody (1∶200, Millipore, MAB3424) and the MF20 antibody (1∶500, Developmental Studies Hybridoma Bank) diluted in Blocking solution 2 as described above.

### Vitelline vessel ligations

HH Stage 18 embryos were windowed and ∼5 ml of albumin was removed from the egg with an 18 gauge needle. An incision was then made through the vitelline and amniotic membranes from the tail bud to approximately the third pharyngeal arch. 10-0 Nylon black monofilament suture (Sharpoint, AA-2510N) was threaded underneath both right vitelline vessels and tied off (Fig. 5B). Successful vascular ligation was confirmed visually by noting that blood flow through the vessels had stopped. Sham operated embryos underwent the same procedure, however, the suture was left in place and not tied off (Fig. 5A). Eggs were then resealed with parafilm and incubated as described above until E6 for optical mapping.

## Supporting Information

S1 Figure Distribution of *Cx40* and *Nav1.5* mRNA in the HH Stage 29 ventricles. **A**) *In situ* hybridization for *Cx40* in a coronal section through a HH Stage 29 heart. **B,C**) Higher magnification images of the areas outlined in (A). Note that *Cx40* shows higher expression in the trabeculae relative to the compact myocardium (red bracket). **D**) *In situ* hybridization for *Nav1.5* in a coronal section through a HH Stage 29 heart. **E,F**) Higher magnification images of regions from (D). As with *Cx40*, *Nav1.5* is enriched in the trabeculae relative to the compact myocardium (red bracket). RA – right atria, LA – left atria, RV – right ventricle, LV – left ventricle, IVS – interventricular septum.
(TIF)

S2 Figure sMHC localization in atrial muscle bundles. **A**) MF20 (red) staining of cardiac muscle in a coronal section through a HH Stage 44 heart. **B**) Confocal image of the boxed atrial region in (A). **C**) Sister section of (B) stained for sMHC (green). Note the periarterial (white arrow) and subendocardial (red arrows) distribution along the atrial muscle bundle. **D**) Confocal image of the boxed ventricular region in (A). **E**) Sister section of (D) stained for sMHC (green). sMHC is expressed in the periarterial (white arrows) and subendocardial (red arrows) Purkinje fibers. RA – right atria, LA – left atria, RV – right ventricle, LV – left ventricle, IVS – interventricular septum.
(TIF)

S3 Figure Vascular remodeling post vitelline vessel ligation. **A**) Bright field image of a sham operated embryo 24 hrs after manipulation. **B**) Bright field image of an embryo 24 hrs post vitelline vessel ligation (blue arrowhead denotes ligation suture). Note the extraembryonic vasculature on the right side of the embryo has regressed. **C**) Bright field image of an embryo 72 hrs post vitelline ligation (blue arrowhead denotes ligation suture). Note that the extraembryonic vasculature on the right side of the embryo has recovered. **D**) Higher magnification image from (C). The blood flow has remodeled around the ligation site.
(TIF)

S1 Movie Optical mapping of the superior surface of a HH Stage 18 atria. Data is presented as the first derivative of the raw acquisition (dV/dT).
(MOV)

S2 Movie Optical mapping of the superior surface of a HH Stage 24 atria. Data is presented as the first derivative of the raw acquisition (dV/dT).
(MOV)

S3 Movie Optical mapping of the superior surface of a HH Stage 29 atria. Data is presented as the first derivative of the raw acquisition (dV/dT). Note that propagation is not uniform, with rapid conduction evident along the bundles of the right atria.
(MOV)
