# Supplementary figures and images for: Hemodynamic Forces Regulate Developmental Patterning of Atrial Conduction
Source: PLoS One. 2014 Dec 12;9(12):e115207. doi: 10.1371/journal.pone.0115207 (PMC4264946; doi:10.1371/journal.pone.0115207)

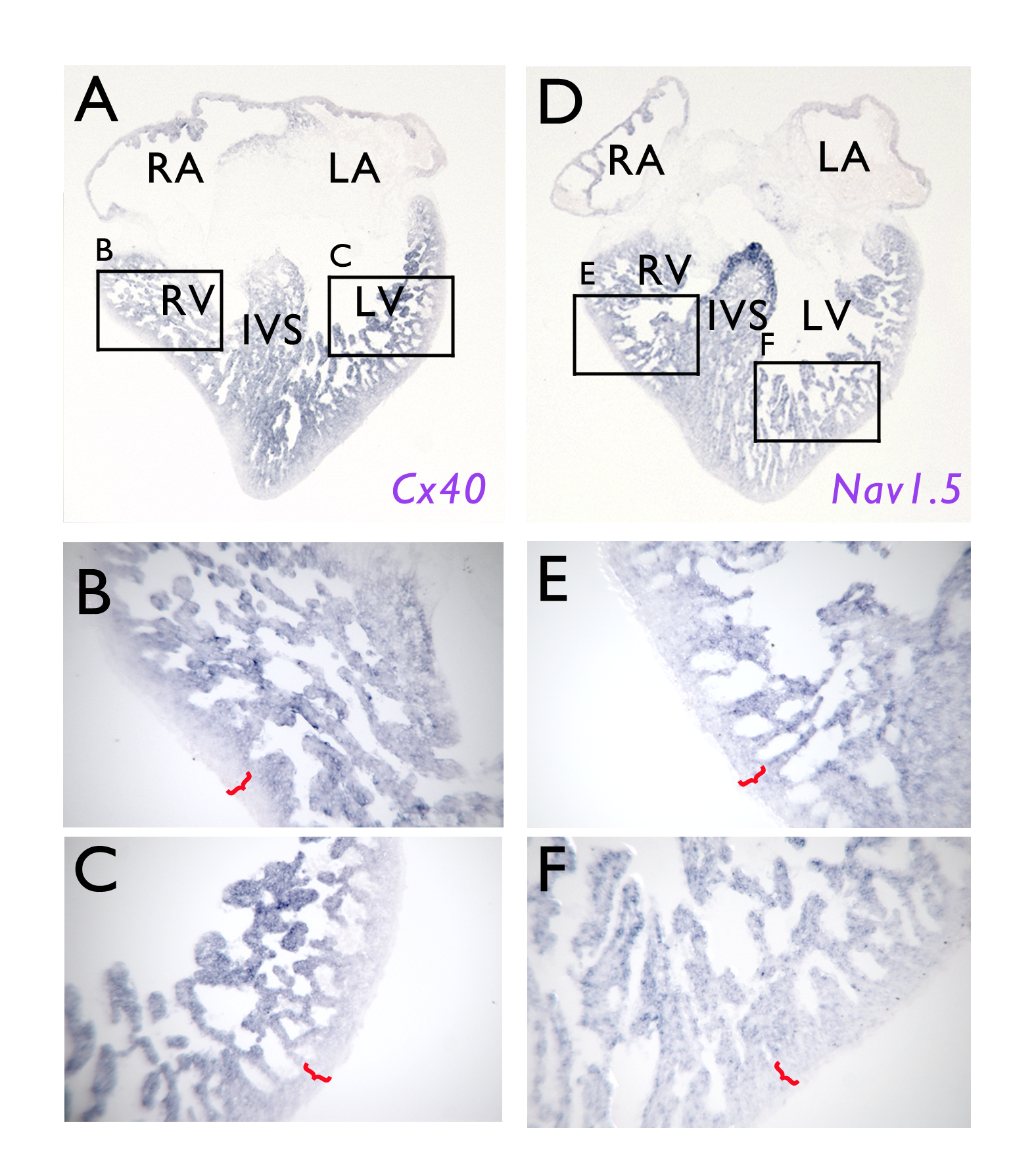

Supplement: S1 Figure — Distribution of Cx40 and Nav1.5 mRNA in the HH Stage 29 ventricles. A) In situ hybridization for Cx40 in a coronal section through a HH Stage 29 heart. B,C) Higher magnification images of the areas outlined in (A). Note that Cx40 shows higher expression in the trabeculae relative to the compact myocardium (red bracket). D) In situ hybridization for Nav1.5 in a coronal section through a HH Stage 29 heart. E,F) Higher magnification images of regions from (D). As with Cx40, Nav1.5 is enriched in the trabeculae relative to the compact myocardium (red bracket). RA – right atria, LA – left atria, RV – right ventricle, LV – left ventricle, IVS – interventricular septum. (TIF) [file pone.0115207.s001.tif]

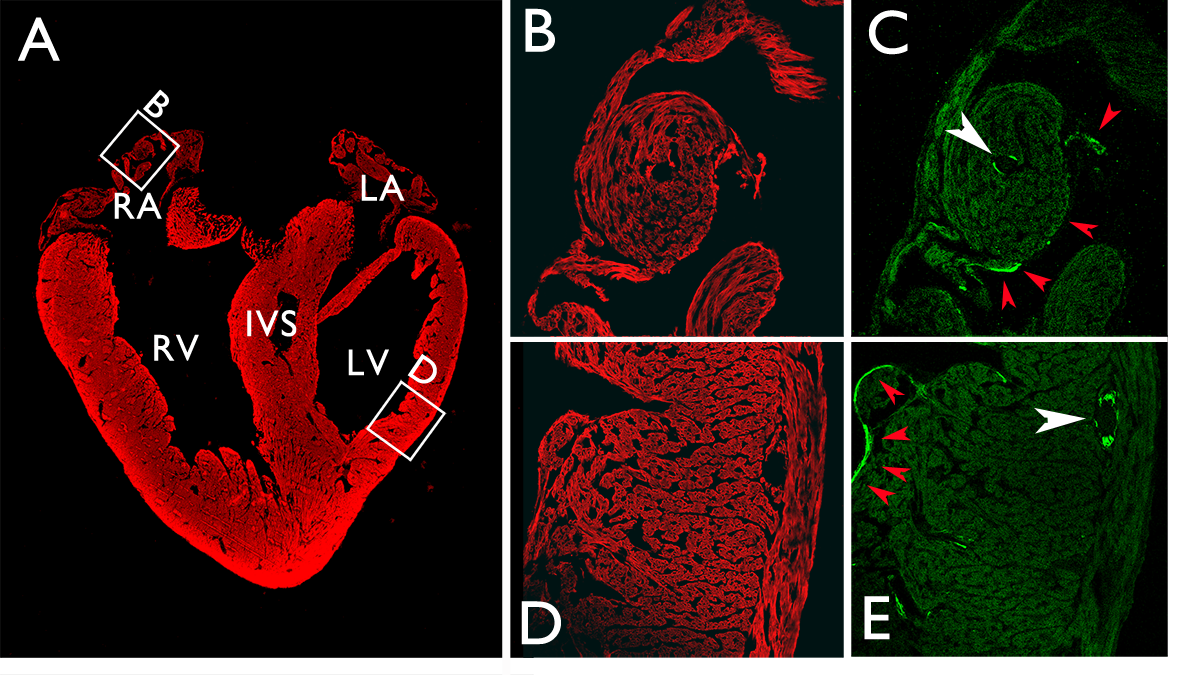

Supplement: S2 Figure — sMHC localization in atrial muscle bundles. A) MF20 (red) staining of cardiac muscle in a coronal section through a HH Stage 44 heart. B) Confocal image of the boxed atrial region in (A). C) Sister section of (B) stained for sMHC (green). Note the periarterial (white arrow) and subendocardial (red arrows) distribution along the atrial muscle bundle. D) Confocal image of the boxed ventricular region in (A). E) Sister section of (D) stained for sMHC (green). sMHC is expressed in the periarterial (white arrows) and subendocardial (red arrows) Purkinje fibers. RA – right atria, LA – left atria, RV – right ventricle, LV – left ventricle, IVS – interventricular septum. (TIF) [file pone.0115207.s002.tif]

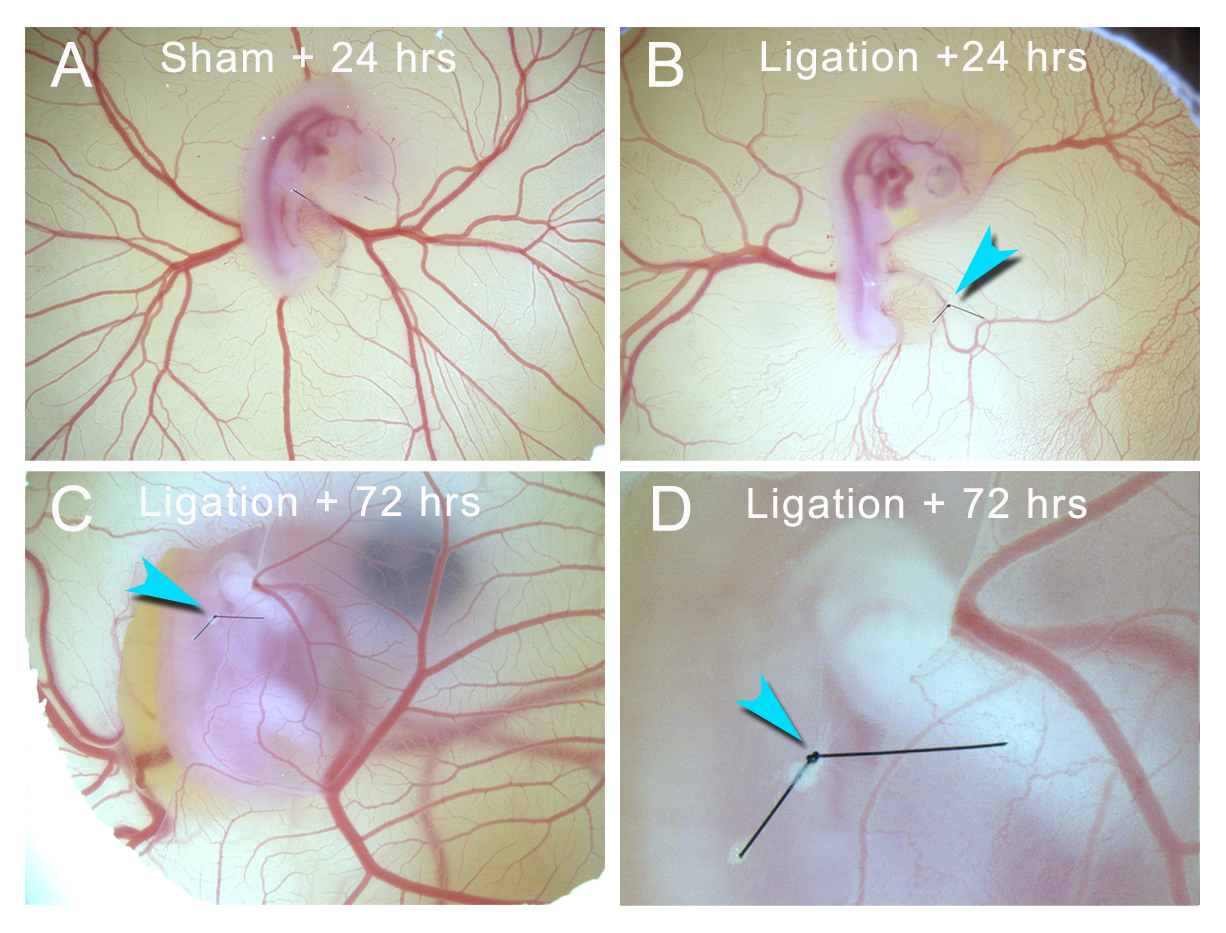

Supplement: S3 Figure — Vascular remodeling post vitelline vessel ligation. A) Bright field image of a sham operated embryo 24 hrs after manipulation. B) Bright field image of an embryo 24 hrs post vitelline vessel ligation (blue arrowhead denotes ligation suture). Note the extraembryonic vasculature on the right side of the embryo has regressed. C) Bright field image of an embryo 72 hrs post vitelline ligation (blue arrowhead denotes ligation suture). Note that the extraembryonic vasculature on the right side of the embryo has recovered. D) Higher magnification image from (C). The blood flow has remodeled around the ligation site. (TIF) [file pone.0115207.s003.tif]
